# *OsNPR1* Enhances Rice Resistance to *Xanthomonas oryzae* pv. *oryzae* by Upregulating Rice Defense Genes and Repressing Bacteria Virulence Genes

**DOI:** 10.3390/ijms24108687

**Published:** 2023-05-12

**Authors:** Xing Dai, Yankai Wang, Kaili Yu, Yonghui Zhao, Langyu Xiong, Ruozhong Wang, Shengben Li

**Affiliations:** 1 Hunan Provincial Key Laboratory of Phytohormones and Growth Development, Hunan Agricultural University, Changsha 410128, China; 2 State Key Laboratory of Crop Genetics and Germplasm Enhancement and Utilization, Nanjing Agricultural University, Nanjing 210095, China; 3 Academy for Advanced Interdisciplinary Studies, Nanjing Agricultural University, Nanjing 210095, China; 4 Institute of Advanced Studies in Humanities and Social Sciences, Beijing Normal University, Zhuhai 519087, China

**Keywords:** dual RNAseq, *OsNPR1*, plant defense, virulence gene, *Xanthomonas oryzae* pv. *oryzae*

## Abstract

The bacteria pathogen *Xanthomonas oryzae* pv. *oryzae* (*Xoo*) infects rice and causes the severe disease of rice bacteria blight. As the central regulator of the salic acid (SA) signaling pathway, NPR1 is responsible for sensing SA and inducing the expression of pathogen-related (PR) genes in plants. Overexpression of *OsNPR1* significantly increases rice resistance to *Xoo*. Although some downstream rice genes were found to be regulated by *OsNPR1*, how *OsNPR1* affects the interaction of rice-*Xoo* and alters *Xoo* gene expression remains unknown. In this study, we challenged the wild-type and *OsNPR1-OE* rice materials with *Xoo* and performed dual RNA-seq analyses for the rice and *Xoo* genomes simultaneously. In *Xoo*-infected *OsNPR1-OE* plants, rice genes involved in cell wall biosynthesis and SA signaling pathways, as well as PR genes and nucleotide-binding site-leucine-rich repeat (NBS-LRR) genes, were significantly upregulated compared to rice variety TP309. On the other hand, *Xoo* genes involved in energy metabolism, oxidative phosphorylation, biosynthesis of primary and secondary metabolism, and transportation were repressed. Many virulence genes of *Xoo*, including genes encoding components of type III and other secretion systems, were downregulated by *OsNPR1* overexpression. Our results suggest that *OsNPR1* enhances rice resistance to *Xoo* by bidirectionally regulating gene expression in rice and *Xoo*.

## 1. Introduction

Rice is one of the most important staple crops in the world. Bacteria blight is a devastating disease of rice and causes 20–50% losses in yield, depending on planting areas and climate conditions [1]. The causative agent of the disease is the Gram-negative bacterium *Xanthomonas oryzae* pv. *oryzae* (*Xoo*) [2]. During infection, bacteria deliver diverse virulence factors to host cells and cause disease. Plants have also evolved resistance genes to overcome the virulence of bacteria and limit their spread. Information exchange and molecular interactions are the foundations for compatibility between pathogens and plant hosts. Over the past few decades, the interaction between *Xoo* and rice has been extensively studied, and some rice cultivars resistant to *Xoo* have been identified. 

A number of factors have been found to be important to *Xoo* virulence. Effectors are conserved virulence factors of bacterial pathogens. Among them, type III secretion (T3S) effectors are best understood. T3S effectors are secreted by bacteria through the T3S system and translocate into plant cells to promote bacterial growth and repress plant defense responses. AvrXa7, AvrXa10, and AvrXa27 of *Xoo* are transcription activator-like (TAL) T3S effectors, and they were found to bind double-stranded DNA and dimerize in the plant cell, which are typical characteristics of many transcription factors [3]. The non-TAL group of T3S effectors was also found to be important for *Xoo* pathogenesis through interactions with rice proteins [4]. T3S is a multi-component protein complex that forms transmembrane channels and ensures the translocation of T3S effectors from bacteria to plant cytoplasm [5,6]. T3S components are encoded by *hypersensitive response and pathogenicity* (*Hrp*) genes. *hrpA*, *hrpB*, *hrpC*, and *hrpD* genes are organized in a 12.2 kb cluster region in the *Xoo* genome, and the mutation of these genes abolished the ability of *Xoo* to elicit disease in rice [7]. In addition to effectors and the T3S system, other virulence factors also contribute to host specificity and bacterial pathogenesis. A mutation in the flagella gene *flhF* of *Xoo* resulted in weak chemotaxis. Extracellular enzymes involved in cell wall degradation, proteolytic, lipolytic, and amylolytic functions were also found to act as virulence factors for *Xoo* and other bacterial pathogens [8,9]. Secretome analysis revealed 109 *Xoo* proteins in the extracellular space of rice leaves. Whether these proteins affect host specificity or pathogenesis remains unclear [1].

To avoid or reduce damage caused by pathogen attacks, plants evolved two layers of their innate immune system. Pathogen-associated molecular pattern-triggered immunity (PTI) recognizes various pathogen-derived molecules, or pathogen-associated molecular patterns (PAMPs), and triggers plant defense responses. Fragments of cell surface macromolecules typical of microorganisms, such as cell wall polysaccharides, secreted proteins, and a flagella protein, often serve as potent PAMPs to induce PTI [10]. PAMPs could be recognized by the pattern recognition receptors (PRRs) located on the cell membrane of plant cells and trigger downstream defense signaling mediated by MAPK cascades and ROS outbursts to inhibit bacteria multiplication in plants [11]. To overcome the basal defense induced by PTI, bacteria deliver effector proteins into plant cells through the T3S system. These effector proteins target various processes in the host cell, like RNA metabolism, the secretion of proteins, and the activation of kinases, to repress PTI [12]. Some effectors could be recognized by plant resistance proteins (R proteins) and trigger the effector-triggered immunity (ETI) of plants. Most of the R proteins belong to the NBS-LRR family [13]. To date, 29 R genes have been identified to confer resistance to rice bacteria blight, of which six have been cloned: *Xa1*, *Xa3*/*Xa26*, *xa5*, *xa13*, *Xa21*, and *Xa27* [14,15,16].

Both PTI and ETI induce systemic immunity in distal tissues, a mechanism termed systemic acquired resistance (SAR) [17]. As an indispensable phytohormone, salicylic acid (SA) is required for plant SAR [18]. The transduction of the SA signal is largely dependent on the *NONEXPRESSOR OF PATHOGENESIS-RELATED GENES 1* (*NPR1*). In uninfected plant cells, NPR1 proteins form oligomers and exist in the cytoplasm; in infected cells, SA induces NPR1 de-oligomerization to form monomers by association with thioredoxins (TRXs) [19]. Free NPR1s move into the nucleus and interact with TGA transcription factors to activate a wide range of gene expressions, including most PR genes [20,21]. NPR1 was found to be required for both PTI and ETI, and the Arabidopsis *npr1* mutant was insensitive to SA treatment [22]. RNAi of *OsNPR1* resulted in reduced resistance to various pathogens, and overexpression of *OsNPR1* significantly enhanced the resistance of rice plants to *Xoo* [23,24].

Interactions between pathogens and plant hosts have been the central question of plant defense studies. During infection, both pathogen and plant genes are dynamically affected by each other. The regulation of plant gene expression under pathogen invasions has been well studied in the past decades. In contrast, how plants affect pathogen gene expression is largely unknown. The synthetic mediums XOM1 and XVM2 could mimic the compositions of plant cell extracts and activate bacterial *Hrp* gene expression [25,26]. However, the metabolites in plant cells are very complicated, and their compositions fluctuate with changes in growth status and environmental conditions. Thus, synthetic media cannot authentically mirror the environment of plant cells. A recent study supplemented rice leaf extract (RLX) into the bacterial medium and found that hundreds of *Xoo* genes were induced by RLX [27]. However, plant cells are highly organized, and each step of signaling transductions or biochemical reactions is under precise orchestration in certain subcellular compartments. Therefore, RLX hardly reflects the real situation in plant cells either. In this study, we inoculated wild type and *OsNPR1* overexpression rice plants with the *Xoo* Philippine Race 6 (PXO99A) strain and performed transcriptome analysis on the *Xoo* and rice genomes simultaneously. Rice genes involved in plant defense were found to be significantly upregulated, and bacteria virulence genes were downregulated by *Xoo* infection in *OsNPR1* overexpression plants, suggesting the function of *OsNPR1* for bidirectional regulation of gene expression in rice and *Xoo*.

## 2. Results

### 2.1. Dual RNAseq Was Developed to Evaluate the Effects of OsNPR1 on Gene Expression of Rice and Xoo

**Figure 1:**
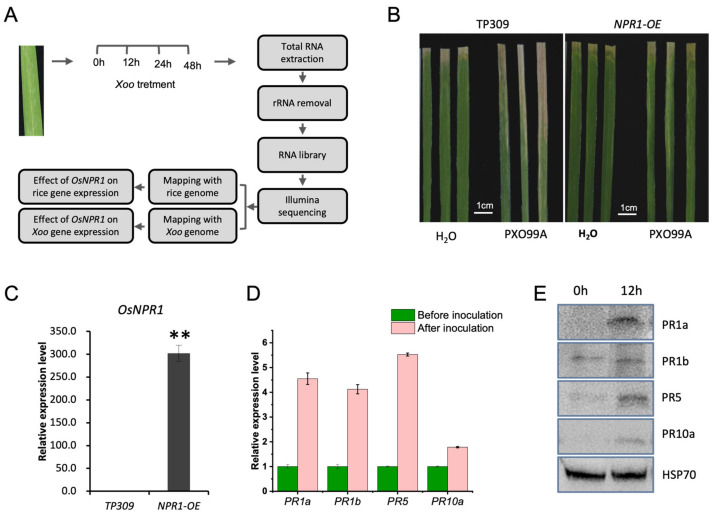
Experiment design and the effectiveness of PXO99A infection: (**A**) Schematic diagram of the dual RNAseq experiment; (**B**) The symptoms of TP309 and *OsNPR1-OE* before and after PXO99A inoculation with leaf clipping. H_2_O in place of PXO99A was used as a control treatment; (**C**) Quantification of *OsNPR1* expression by real-time PCR. Asterisk indicates statistically significant differences between TP309 and *OsNPR1-OE* (** *p* < 0.01; Student’s *t*-test); (**D**) Quantification of PR gene expressions in *OsNPR1-OE* by real-time PCR before and after PXO99A inoculation. *OsActin* was used as the internal control; (**E**) Examination of PR proteins in *OsNPR1-OE* before and after PXO99A inoculation by western blot. Hsp70 was used as an internal control.

TP309 is a rice japonica variety without *Xa21* and is susceptible to PXO99A [28]. As illustrated in Figure 1A, leaves of TP309 and *OsNPR1-OE* were inoculated with the PXO99A strain, and RNAs were isolated from the time points of 0, 12, 24, and 48 h after inoculation. As polyadenylation does not occur on bacterial RNAs, we performed RNAseq using RNAs after rRNA removal. The reads were mapped to the rice and PXO99A genomes simultaneously to assess the effects of *OsNPR1* on rice and bacterial gene expression. To ensure that our treatment is effective and the materials are correct, we first infected TP309 and *OsNPR1-OE* with PXO99A by leaf clipping. As expected, TP309 is susceptible to the strain; the lesions expanded rapidly on leaves after *Xoo* inoculation compared to those of the H_2_O treatment. In contrast, *OsNPR1-OE* plants displayed enhanced resistance, and only small areas of the leaves showed disease symptoms (Figure 1B). We also checked the expression of *OsNPR1* in the two genotypes. The expression is much higher in the *OsNPR1-OE* lines than in TP309, demonstrating that the transgene is not silenced (Figure 1C). After inoculation, four PR genes associated with Xoo resistance were significantly increased at both RNA and protein levels in *OsNPR1-OE* leaves compared to TP309 (Figure 1D,E).

**Figure 2:**
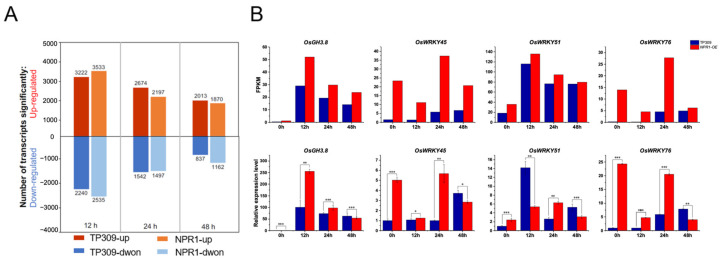
DEGs of RNAseq and validation of selected genes in RNAseq data: (**A**) DEGs between TP309 and *OsNPR1-OE* at different time points after PXO99A inoculation. The number of DEGs was labeled at the top or beneath the corresponding bars. (**B**) Expression validation of selected genes from RNAseq data by real-time PCR. The top graphs are the FPKM values of each selected gene in TP309 and *OsNPR1-OE* at different time points after PXO99A inoculation, and the bottom ones are the relative expression of the corresponding genes examined by real-time PCR. *OsActin* was used as the internal control. T represents TP309 and N represents *OsNPR1-OE*, and the number after T or N represents the time points after PXO99A inoculation. Statistical differences were examined by the student’s *t*-test. *, **, and *** mean *p* ≤ 0.05, *p* ≤ 0.01, and *p* ≤ 0.001, respectively.

A total of 18116 genes were detected and mapped to the rice genome. Thousands of differentially expressed genes (DEGs) between TP309 and *OsNPR1-OE* at different time points after inoculation were found (Figure 2A). To verify the reliability of the RNAseq data, we chose four genes that had been reported to be involved in *Xoo* resistance in rice and validated the RNAseq results by real-time PCR. Among these, the expression of *OsGH3.8* was found to be regulated by *OsNPR1*, and three *WRKY* genes encode downstream transcription factors of SA signaling [29,30,31]. The overall expression patterns of these four genes at various time points were highly consistent with the results of RNAseq, although there were some variations between the two sets of data that might result from the independent pathogen treatment on rice for each experiment (Figure 2B).

### 2.2. Genes Involved in Cell Wall Biosynthesis and SA Signaling Pathways Were Upregulated by OsNPR1 Overexpression after PXO99A Infection

**Figure 3:**
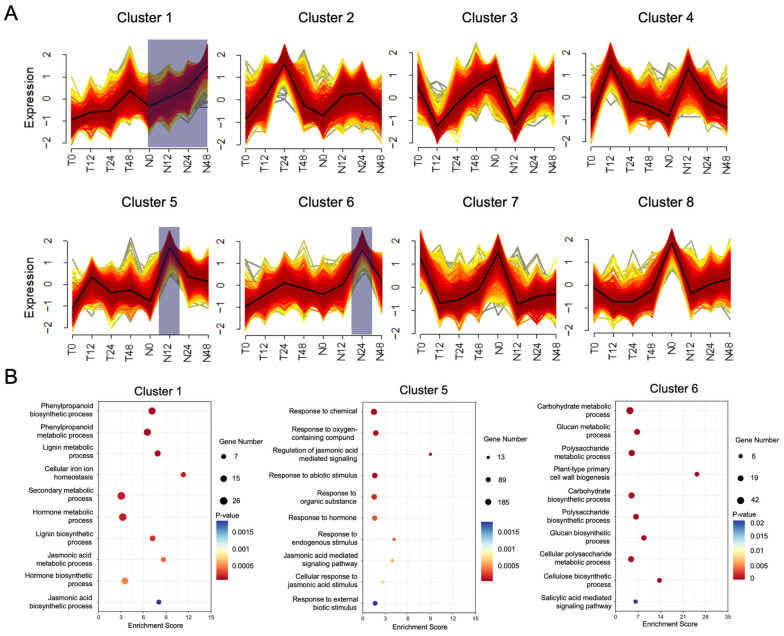
Gene clustering by the expression profiles in TP309 and *OsNPR1-OE*: (**A**) Fuzzy c-means clustering based on different expression profiles of all detected genes from TP309 and *OsNPR1-OE* at different time points after PXO99A inoculation. The altered profiles between TP309 and *OsNPR1-OE* in clusters 1, 5, and 6 were highlighted in light blue. (**B**) GO analyses of genes in clusters 1, 5, and 6.

As a master regulator of plant disease resistance, overexpression of the NPR1 gene has been demonstrated to enhance plant diseases in different plant species. To understand the mechanism by which *OsNPR1* regulates rice resistance to Xoo, we globally compared the time course expression of rice genes between TP309 and *OsNPR1-OE* after Xoo inoculation. Eight different clusters were obtained from the analysis (Figure 3A). Among them, clusters 1, 5, and 6 displayed distinct patterns between TP309 and *OsNPR1-OE* (Table S1). The expression of cluster 1 genes increased slowly in TP309 over the time after inoculation but increased more rapidly in *OsNPR1-OE* plants (Figure 3A). In this cluster, genes in phenylpropanoid and lignin biosynthetic pathways were highly enriched (Figure 3B, left). Three phenylalanine ammonia lyase (PAL) genes (*Os04g43760*, *Os02g41630*, and *Os04g43760*) and one caffeic acid 3-O-methyltransferase (COMT) gene (*Os12g13810*) were included in this cluster.

The expression of genes in cluster 5 increased rapidly in the first 12 h, then decreased gradually. Both TP309 and *OsNPR1-OE* showed similar patterns, with the highest value at the time point of 12-h, but the peak of *OsNPR1-OE* was much higher than that of TP309 (Figure 3B, middle). Genes in the JA signaling pathway were enriched in this cluster, including four genes encoding JAZ domain-containing proteins (Os03g08320, Os03g08310, Os10g25290, and Os10g25230). Genes involved in the response to external biotic stimuli were also enriched in this cluster. These genes encode a BRI1-like protein (Os08g23290), a multidrug resistance-associated protein (MRP/ABCC, Os04g13210), an ethylene-responsive transcription factor ERF110 (Os11g06770), and three WRKY transcription factors (WRKY16/Os01g47560, WRKY11/Os01g43650, and WRKY49/Os05g49100).

The expression profiles of genes in cluster 6 were similar to those in cluster 5, except that the peak was reached at the 24-h time point (Figure 3A). Genes involved in the SA signaling pathway were significantly enriched in this cluster, including four *WRKY* genes (*WRKY45/Os05g25770*, *WRKY74/Os09g16510*, *WRKY69/Os08g29660*, and *WRKY76/Os09g25060*), *OsNPR3*(*Os03g46440*), and two *Glutaredoxin* (*GRX*) genes (*Os05g48930* and *Os01g47760*). Another type of highly enriched gene in cluster 6 was related to plant cell wall biosynthesis. These included genes encoding for six cellulose synthases (CESA1/Os05g08370, CESA2/Os03g59340, CESA3/Os07g24190, CESA4/Os01g54620, CESA6/Os07g14850, and CESA8/Os07g10770), a cellulose synthase-like protein (CSLE1/Os09g30120), two COBRA proteins (Os05g32110 and Os03g54750), and other glycosyl transferases (Os03g11330 and Os07g41360) (Figure 3B, right).

### 2.3. OsNPR1-Enhanced Expression of PR Genes and R Genes after PXO99A Infection

**Figure 4:**
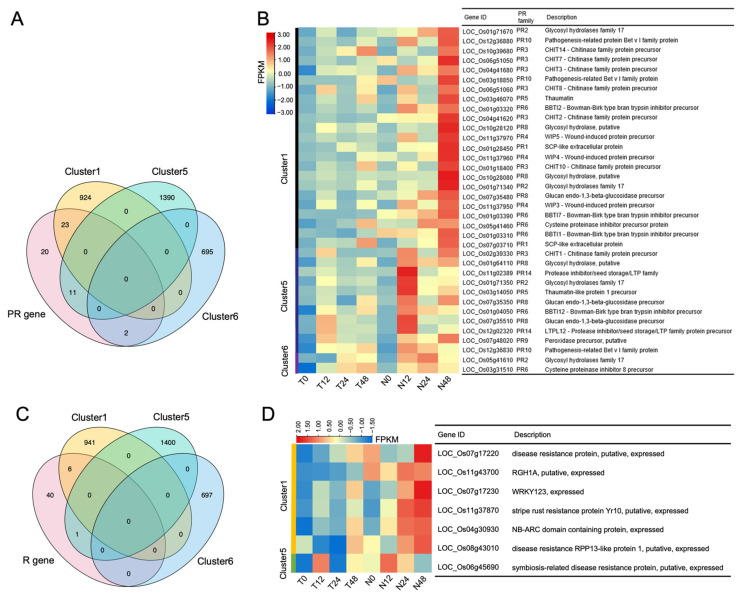
Differentially expressed PR and R genes between TP309 and *OsNPR1-OE*: (**A**) Venn diagram of the differentially expressed PR genes and the three gene clusters. PR genes with FPKM value ≥ 1 are displayed on the diagram; (**B**) A heat map and the corresponding gene IDs of the differentially expressed PR genes; (**C**) A venn diagram of the differentially expressed R genes and the three gene clusters. The R genes with FPKM values ≥ 1 are displayed on the diagram; (**D**) Heat map and the corresponding gene IDs of the differentially expressed R genes.

As the master regulator of SA-mediated SAR, NPR1 is necessary for the expression of PR genes. The rice genome was annotated to contain 113 PR genes belonging to 12 families [32]. To reveal the regulation of PR genes by *OsNPR1*, we investigated the expression of all 113 genes in our RNAseq data. A total of 36 PR genes fell into clusters 1, 5, and 6 (Figure 4A). 23 PR genes were found in cluster 1. In TP309, the expressions of the cluster 1 genes were very low at the 0-h time point and increased gradually to a medium level with infection by PXO99A, while in *OsNPR1-OE* plants, their expressions were induced more rapidly and reached a much higher level 48 h after inoculation. The PR genes in cluster 1 belonged to PR gene families 1–6, and 8–10, respectively. 11 PR genes were found in cluster 5, and they were barely detectable at the 0-h time point for both TP309 and *OsNPR1-OE* plants. In TP309, their expressions were induced to a medium level with prolonged time after inoculation and reached their peak at 48 h. In *OsNPR1-OE* plants, the gene expressions were rapidly induced and accumulated to a maximum level 12 h after inoculation. The PR genes of cluster 5 belonged to PR families 2, 3, 5, 6, 8–10, and 14, respectively. Only two PR genes were found in cluster 6, with relatively low expressions, and they were more rapidly induced by PXO99A in *OsNPR1-OE* (Figure 4B).

R proteins are crucial for plant susceptibility as well as the resistance strength of plants to various pathogens. Global prediction identified 332 NBS-LRR genes in rice [33]. After removing duplicate genes, we recovered 132 unique gene IDs (Table S2). A total of seven R genes fell into cluster 1 and cluster 5 (Figure 4C). Six R genes were found in cluster 1, with dramatic induction by PXO99A infection in *OsNPR1-OE* plants compared to TP309. One R gene was found in cluster 5, with a relatively higher expression level in *OsNPR1-OE* at all time points (Figure 4D).

### 2.4. Expression of Genes Involved in Cellular Metabolisms of PXO99A Was Downregulated by OsNPR1

**Figure 5:**
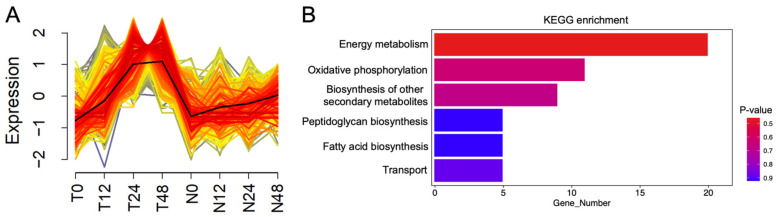
A cluster of *Xoo* genes with altered expression profiles between TP309 and *OsNPR1-OE*; (**A**) Fuzzy c-means clustering shows the dynamic expression profiles of *Xoo* genes in TP309 and *OsNPR1-OE* at different time points after PXO99A inoculation; The color gradient represents the relationship of the genes to the cluster, with red and grey indicating the closest and furthest relationship, respectively. (**B**) KEGG analysis of all the genes in this cluster.

In order to assess the effects of *OsNPR1* on host virulence gene expression, we mapped the RNAseq reads to the PXO99A genome. A total of 3521 genes were detected and mapped to the PXO99A genome, and one cluster of genes displayed distinct expression patterns between TP309 and *OsNPR1-OE* plants (Figure 5A, Table S3). In TP309, the expression of these genes increased rapidly with prolonged time after PXO99A inoculation; the maximum level was reached at the 24-h time point and maintained thereafter. However, the expression of these genes in *OsNPR1-OE* was significantly lower than that of TP309, and they were barely induced by the infection of PXO99A. KEGG analysis showed that genes involved in pathways of energy metabolism, oxidative phosphorylation, biosynthesis of secondary metabolism, peptidoglycan biosynthesis, fatty acid biosynthesis, and transportation were enriched in this cluster (Figure 5B). Of the genes involved in transport, sodium/glucose cotransport (PXO_RS25965), Mg++/citrate complex transporter (PXO_RS19425), and annotated transporter (PXO_RS07745) were included in this cluster.

### 2.5. Expression of PXO99A Virulence Genes Was Repressed by OsNPR1

**Figure 6:**
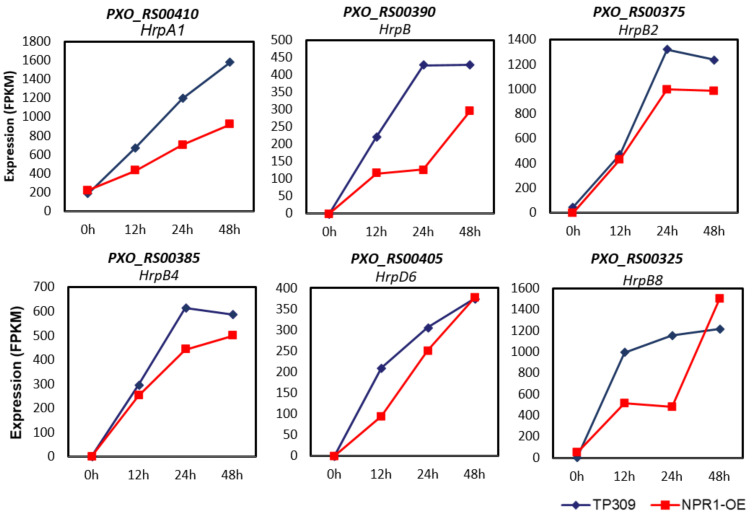
Expression of *Xoo Hrp* genes in TP309 and *OsNPR1-OE* plants. The numbers on the y-axis are the FPKM values of each *Hrp* gene.

To overcome PTI in plants, bacteria inject effector proteins into plant cells via the T3S system, which are encoded by *Hrp* genes. To investigate whether *OsNPR1* could influence the expression of *Xoo Hrp* genes, we checked the expression profiles of all *Xoo hrp* genes in RNAseq data and found six *Hrp* genes (*HrpA1/PXO_RS00410*, *HrpB/PXO_RS00390*, *HrpB2/PXO_RS00375*, *HrpB4/PXO_RS00385*, *HrpB8/PXO_RS00405*, and *HrpD6/PXO_RS00325*) were downregulated in *OsNPR1-OE* plants after PXO99A infection, indicating that *OsNPR1* might restrict the delivery of *Xoo* effectors into plant cells by repressing *Hrp* gene expression (Figure 6).

**Figure 7:**
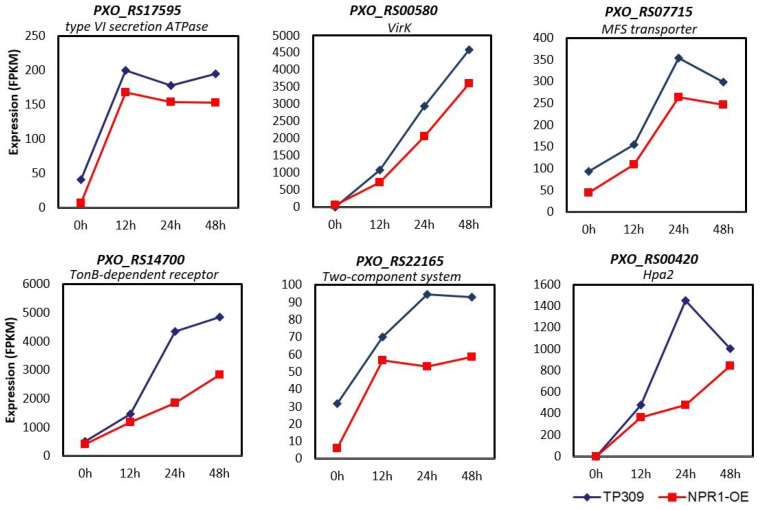
Expression of virulence genes other than *Hrp* in TP309 and *OsNPR1-OE*. The numbers on the y-axis are the FPKM values of each virulence gene.

In addition to *Hrp* genes, the expression of other virulence genes was also examined in our RNAseq data. Six virulence genes were revealed to be downregulated in *OsNPR1-OE* plants (Figure 7). Among them, *PXO_RS17595* encodes a type VI secretion ATPase; *PXO_RS00580* encodes virK, a periplasmic protein that is required for some toxin secretion; *PXO_RS07715* encodes a major facilitator superfamily (MFS) transporter; *PXO_RS14700* encodes a TonB-dependent receptor; *PXO_RS22165* encodes a two-component system protein; and *PXO_RS00420* encodes a Hpa2 translocator of the T3S system. The downregulation of these important virulence genes indicates that *OsNPR1* may enhance rice resistance to *Xoo* by repressing virulence gene expression of the pathogen.

We chose several genes and validated their expression through real-time PCR and found that the results were consistent with the RNAseq data, suggesting the reliability of the gene expression profiles by RNAseq analyses (Figure S1).

## 3. Discussion

The interactions between plant host and pathogen are central questions in plant immunity studies. As the opposite sides of combat, plants and pathogens fight each other for survival and have co-evolved sophisticated interactive mechanisms. Bacterial pathogens deliver various forms of virulence factors into plant cells, and the plant immune system employs PTI and ETI in a “zigzag” mode to restrict the spread of pathogens. The molecular interactions between plant and host determine the susceptibility and resistance of the host plants. SA-mediated SAR endows the plant with long-lasting and enforced disease resistance. NPR1 serves as the master regulator of SA signaling transduction, and overexpression of *NPR1* genes results in dramatically enhanced disease resistance in various plant species. Several studies have revealed differentially expressed genes in *OsNPR1* knockdown or overexpression plants. However, whether *OsNPR1* can regulate the expression of *Xoo* genes remains unknown. In this study, we conducted dual RNAseq analyses on POX99A-treated rice leaves from TP309 and *OsNPR1-OE* plants and found that *OsNPR1* enhanced rice resistance to *Xoo* by increasing the expression of plant defense genes and, on the other hand, repressing the expression of host virulence genes. These results indicate the complicated functions of *OsNPR1*, particularly its role in regulating the genes of invading pathogens.

### 3.1. Dual RNAseq Is an Excellent Strategy for Simultaneous Analyses of the Gene Expression of Interactive Organisms

The expression of plant defense genes and pathogen virulence genes is dynamically regulated during infection. For instance, bacterial *Hrp* genes were expressed at a very low basal level prior to infection but rapidly induced after invading plant cells [34]. A number of studies have been conducted on the plant genes induced by pathogen infections. However, it is difficult to investigate the effects of plant factors on pathogen gene expression because of the relatively low accumulation of pathogens in infected plant cells. By supplementing with fructose or xylose, the synthetic medium XVM2 or XOM2 can mimic the apoplast environment of plants to some extent and facilitate the identification of many *Hrp* and other virulence genes of bacterial pathogens [35,36]. The compositions of plant cells and apoplasts are complicated and fluctuate frequently in response to environmental stimuli and developmental signals. Therefore, synthetic mediums cannot reflect the in vivo situations of plant cells. To overcome this disadvantage, Kim et al. substituted rice leaf extract (RLX) for sugars used in XVM2 and XOM2 mediums and revealed expression profiles of *Xoo* genes induced by RLX in a time-course manner [27]. Although this method included the full composition of the plant cell metabolites in the medium, it abolished the plant cell compartments where cellular metabolism and signal transduction take place. Both approaches are in vitro systems, but the growth of bacteria in medium is largely different from that in live plant tissues. In this study, we inoculated *Xoo* on rice leaves and conducted the dual RNAseq analyses for the rice and POX99A genomes simultaneously. Compared to XVM2/XOM2 and RLX mediums, our system in vivo and the gene expression data may more authentically reflect the real responses of *Xoo* and rice during plant-pathogen interaction.

Several aspects need to be taken into consideration for this approach. Firstly, bacteria mRNAs lack the polyA modification at the 3′ terminus, so oligo(dT) beads are not suitable for mRNA purification in this case. We utilized the rRNA removal method to enrich mRNAs from both rice and *Xoo*. Secondly, considering the small portion of *Xoo* biomass in comparison to that of rice in the infected leaves, a higher sequencing depth is required to detect enough *Xoo* RNAs. In this study, we obtained 12 G reads from the RNAseq data. According to the *Xoo* genome project, there are 3952 annotated coding genes in total. Our RNAseq data resolved 3521 genes with FPKM values larger than 1, accounting for 89% of all annotated *Xoo* genes. At the same time, we detected 18,116 out of the total 55,985 annotated rice genes (Figure 2A). Thousands and hundreds of genes from rice and *Xoo* were found to be differentially expressed between TP305 and *OsNPR1-OE* plants, suggesting sufficient information in the data.

### 3.2. OsNPR1 Enhances Rice Resistance to POX99A by Upregulating the Expression of Rice Genes Involved in Multi-Layer Defense Responses

Plants prevent the invasion and spread of pathogens through multiple strategies. The first defense barrier is the plant cell wall. In cluster 1, genes in the lignin biosynthetic pathway were enriched, including three *PAL* genes and one *COMT* gene, which are important enzymes for lignin synthesis (Figure 3B, left). Lignin is a hydrophobic phenylpropanoid polymer formed by the oxidative polymerization of three main monolignols. As an important composition in the secondary cell wall, lignin cross-links with other cell wall components such as polysaccharides and proteins to form network structures [37]. Lignification is a common plant response to microbial infection. Upon *Pseudomonas syringae* pv. *tomato* invasion, monolignol polymerization occurred in highly restricted areas around the pathogen cells, and localized lignification surrounding the bacteria reduced their movement [38]. In addition, other compounds generated from the phenylpropanoid pathway, such as phenolic phytoalexins, isoflavans, and isoflavanones, were also implicated in plant defense [39]. Genetic studies revealed that eight *OsPAL* genes were linked to resistance to multiple pathogens in rice [40]. The resistance to rice blight in the *ospal4* mutant was reduced, suggesting a positive role for *OsPAL4* in rice disease resistance [41]. Cellulose is the most abundant polysaccharide in plant cells and constitutes the backbone network of the cell wall. In cluster 6, six *CESA* and one *CSLE*, which are responsible for cellulose synthesis, were significantly upregulated in *OsNPR1-OE* plants after POX99A inoculation (Figure 3B, right). This cluster also included two *COBRA* genes, which facilitated the deposition of cellulose crystals [42]. The other two glycosyl transferases may be involved in the formation of hemicellulose. The upregulation of cell wall-associated genes in *OsNPR1-OE* plants after POX99A infection indicates that *OsNPR1* may enforce cell wall strength and rigidity to restrict the spread of *Xoo*.

SA and JA are two important phytohormones regulating plant defense responses. SA is crucial to plant SAR, and the presence of SA is perceived by NPR1, which in turn binds to TGA transcription factors and activates a series of pathogen-related gene expressions. In addition to TGAs, WRKY transcription factors were also found to be important for SA signal transduction. Several members of the WRKY family members in Arabidopsis have been found to be positive regulators of plant defense. AtWRKY18, 53, 54, and 70 were all positive regulators of SA-mediated defense responses [43,44]. In cluster 5, six *WRKY* genes were upregulated in *OsNPR1-OE* plants after POX99A inoculation. The enrichment of *WRKY* genes in *OsNPR1-OE* indicated their involvement in the *OsNPR1*-mediated plant defense response. Two *GRX* genes were also included in this cluster. The Arabidopsis homolog of *GRX* has been reported to be induced by SA and to play a role in the crosstalk between SA and JA [45]. Interestingly, we found that *OsNPR3* in cluster 5 was upregulated by *OsNPR1-OE* after PXO99A inoculation. NPR3 and NPR4 act as adaptor proteins between NPR1 and CUL3 and facilitate proteolytic degradation of NPR1. The occurrence of *OsNPR3* in cluster 5 indicates a balancing mechanism to prevent the hyperaccumulation of OsNPR1 and reduce its fitness cost [46]. JA is another vital phytohormone for plant defense. The presence of JA is perceived by the SCFCOI1-JAZ co-receptor complex, which leads to the proteolytic degradation of the JAZ transcription repressor and thus promotes downstream gene expression [47]. We found four JAZ domain-containing genes in cluster 5. JA signal pathways were reported to be repressed by SA [17]. The occurrence of these transcription repressors of the JA signal in this cluster was consistent with the negative regulation of SA on JA signaling.

PR genes are a group of genes induced by pathogen invasion, most of which act to repress the multiplication and spread of pathogens. Plant PR genes can be divided into 17 families, and the rice genome was annotated to encode 40 PR genes [32,48]. We systemically analyzed the expression patterns of all of these PR genes in rice and found that members in families 1–6, 8–10, and 14 were upregulated in *OsNPR1-OE* plants after POX99A inoculation (Figure 4B). While not all PR gene functions are well understood currently, it is known that families 1–3 belong to serine carboxypeptidase-like proteins (SCPLs), β-1,3-glucanse, and chitinase, respectively, indicating that *OsNPR1* upregulated the expression of these hydrolysis genes to degrade the composition of *Xoo* cells. Family 5 belongs to thaumatin-like proteins (TLPs), which have anti-fungal activities. Famies 6 and 14 belong to proteinase inhibitors, which may function to inhibit the protease activity of *Xoo* and prevent the degradation of rice proteins. Family 9 belongs to peroxidase, which is involved in lignin biosynthesis. In addition, 14 out of 132 R genes were upregulated in *OsNPR1-OE* plants. These results imply the role of *OsNPR1* in upregulating the expression of rice PR and R genes to enhance resistance to *Xoo*.

### 3.3. OsNPR1 Enhances Rice Resistance to Xoo by Repressing the Expression of Bacteria Virulence Genes

Bacterial pathogens deliver various virulence factors into host cells during infection. Among them, *Hrp* genes are the most important. *Hrp* genes encode the T3S system, through which effector proteins are injected into plant cells. Six *Hrp* genes were found to be downregulated in *OsNPR1-OE* plants after *Xoo* inoculation. All of these genes were induced after inoculation, with maximum expression occurring at various time points. The *Hrp* genes were organized into clusters in *Xoo*. It is interesting to note that most *HrpB* genes reached their highest expression levels at the 24 h time point, and *HrpA1* and *HrpD6* at 48 h (Figure 6). The transcription trends of these *Hrp* genes are consistent with their organization in the genome. *HrpB* genes were localized closely and transcribed with the same orientation, while *HrpA1* and *HrpD6* genes are relatively far away from *HrpBs* and transcribed in opposite directions [49]. In addition, six virulence genes other than *Hrp* were also downregulated in *OsNPR1-OE* plants (Figure 7). Hpa2 is an important component of T3S and Clp V ATPase is a key conserved component in the type VI secretion system, which may provide power for the secretion of substrate proteins [50]. virK is a periplasmic protein that is considered a secreted protein and may play a virulent role associated with the type IV secretion system for some toxin secretion. MFS is a transporter in bacterial metabolism. In *Aspergillus oryzae*, abolishment of the MSF gene *Aokap4* resulted in the loss of the ability to secrete kojic acid [51]. It is worth noting that the expression of all of these virulence genes was induced after inoculation, but different genes reached maximum expression levels at various time points, indicating that virulence genes are regulated by different mechanisms or transcription factors. The downregulation of the expression of these different virulence genes suggests that it may be a conserved strategy of OsNPR1 to choose various *Xoo* virulence genes as the repressing targets to reduce bacteria’s virulence. In addition, OsNPR1 also downregulated a set of genes involved in energy metabolism, biosynthesis of primary and secondary metabolism, and transportation to restrict the growth of *Xoo* in plant cells (Figure 5B).

**Figure 8:**
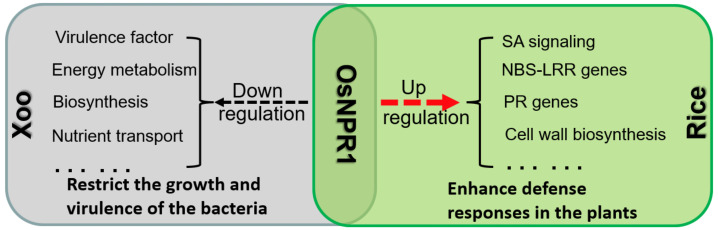
A model for the enhancement of rice resistance to *Xoo* by OsNPR1 bidirectional regulation of gene expression in the host and pathogen.

As the central regulator of SA signaling, the roles of NPR1 in plant disease defense have been substantially studied in different plant species. It is interesting to find that OsNPR1 could bidirectionally affect the expression of genes from rice and *Xoo*. On the one hand, OsNPR1 enhanced the plant defense response by upregulating genes involved in the SA signaling pathway, PR genes, R genes, and genes involved in cell wall biosynthesis in rice. On the other hand, it repressed growth and reduced the virulence of *Xoo* by downregulating the expression of multiple virulence genes and genes involved in energy metabolism, biosynthesis, and nutrient transportation (Figure 8). Whether OsNPR1 directly or indirectly regulates *Xoo* gene expression and which proteins of *Xoo* interact with OsNPR1 require further investigations.

## 4. Materials and Methods

### 4.1. Bacterial Strains, Culture, and Growth Conditions

*Xanthomonas oryzae* pv. *oryzae* strain PXO99A (Philippine race 6, PR6) was grown on nutrient agar (NA) medium plates (0.3% beef extract, 0.1% yeast extract, 0.5% polypeptone, 1% sucrose, 1.5% agar, 100 μg/mL rifampicin, pH 7.0) at 28 °C.

### 4.2. Rice Materials and Growth Conditions

The Japonica rice cultivar Taipei 309 (TP309) and the *OsNPR1* overexpression line (*NPR1-OE*) were grown in a growth chamber for 6 weeks with the temperature at 28 °C during daytime and 23 °C at night. The photoperiod is 16 h light/8 h dark, and the relative humidity is 80%.

### 4.3. Pathogen Inoculation

*Xoo* strain PXO99A was grown on NA medium plates at 28 °C for 2 days, resuspended in sterile water, and diluted to OD_600_ = 0.5 for inoculation. To test the effectiveness of *Xoo* virulence, the second and third leaves of 6-week-old plants were picked and inoculated by the leaf-clipping method [52]. Disease symptoms were scored by measuring the lesion length at 14 days post-inoculation. For RNAseq, the second and third leaves of each plant were inoculated with the *Xoo* suspension via the penetration method using a needleless syringe [53]. The infiltrated regions of leaves were separately collected at 0, 12, 24, and 48 h after inoculation, immediately frozen in liquid nitrogen, and stored at −80 °C.

### 4.4. Quantitative Real-Time PCR

Approximately 100 mg of rice leaf tissue was collected for total RNA extraction with TRIzol (Ambion, Austin, TX, USA). Quantitative real-time PCR was performed using HiScript III RT SuperMix (Vazyme, Nanjing, China). Primers used are listed in Table S4. Rice *OsActin* gene expression was used as the internal control, and relative expression values were calculated using the comparative 2^−ΔΔCt^ method.

### 4.5. Protein Detection by Western Blot

Total proteins were separated using 10% SDS-PAGE and blotted onto polyvinylidene difluoride (PVDF) membranes (Bio-Rad, Hercules, CA, USA). Mouse antibodies against plant PR1a, PR1b, PR5, PR10a, and HSP70 were used at ratios of 1:1000.

### 4.6. RNAseq and Data Process

For RNAseq library construction, rRNAs were removed from total RNAs with the RiboMinus Plant kit (ThermoFisher, Waltham, MA, USA) for mRNA enrichment. The mRNAs were degraded into 300-nt fragments with an RNA fragmentation reagent (Ambion, USA). cDNAs were synthesized by reverse transcription with random hexamer primers. RNAseq libraries were generated using an NEBNext Ultra RNA library prep kit (NEB, Ipswich, MA, USA), and 150-bp pair-end RNA sequencing was conducted using the Illumina NovaSeq 6000 platform at the Personalbio company. Reads of RNAseq data were mapped to the rice genome v7 and the PXO99A genome separately [54]. The significance of differentially expressed genes (DEGs) was determined by a fold change ≥ 2 and a *p*-value < 0.05.

### 4.7. Gene Clustering

Genes detected for TP309 and *OsNPR1-OE* after different time points after PXO99A inoculation were filtered with a CV cutoff (>0.3) and grouped into various clusters using the Mfuzz package with the fuzzy c-means algorithm in R software v4.1.3 [55].

## 5. Conclusions

In this study, we inoculated rice varieties TP309 and *OsNPR1-OE* plants with PXO99A for various periods of time and applied dual RNAseq analyses against the rice and PXO99A genomes simultaneously. OsNPR1 was found to enhance rice resistance to *Xoo* by increasing the expression of rice defense-related genes and repressing *Xoo* virulence genes. These findings expand on the understanding of OsNPR1 function in rice resistance to *Xoo* and will benefit the mechanistic study of OsNPR1-mediated plant defense and the identification of target genes for *Xoo* control.

## Data Availability

All RNAseq data were deposited at BioProject with the accession number PRJNA957574.

## Supplementary Materials

The supporting information can be downloaded at: https://www.mdpi.com/article/10.3390/ijms24108687/s1.
