# Supplementary material for: OsNPR1 Enhances Rice Resistance to Xanthomonas oryzae pv. oryzae by Upregulating Rice Defense Genes and Repressing Bacteria Virulence Genes
Source: Int J Mol Sci. 2023 May 12;24(10):8687. doi: 10.3390/ijms24108687 (PMC10218434; doi:10.3390/ijms24108687)
Supplement: Supplementary file 1 [file ijms-24-08687-s001.zip › Table S4 Primers for real time PCR.pdf]

Table S4. List of the primer pairs of qPCR genes

Rice gene	primer-F	primer-R
OsNPR1	TGAGAGTCTACGAGGAAGGTTGC	CGTTGTCTTTCAGGAGGTGGAT
OsGH3.8	CCGGTCATGAACTTGTATGTGC	CGTGTAGTTGTGGTACGGGT
OsPR10a	CGCCGCAAGTCATGTCCTAA	CTTCGTCTCCGTCGAGTGTG
OsWRKY45	ATTCGGTGGTCGTCAAGAACC	CACCGGAAGTAGGCCTTTG
OsWRKY51	CATCGGGTGAAGAGGACGAT	TAGTATCCTCGTGGGAAGGGG
OsWRKY76	AGCTGCCCGAATTCTAGCTT	GCCCAAGGACCAACAGGTTA
OsPR1a	TATGCTATGCTACGTGTTTATGC	CACTAAGCAAATACGGCTGACA
OsPR1b	ATTGCTTTGGCCATGGTAGC	CGTTGTGGAGCCTCACGTA
OsPR5	GCAACAGCAACTACCAAGTCG	ATGATTATCGATCAAGGTGTCGT
OsActin	TCCGTAAGTACGTGGGGAGT	GCAGACTAGCAGTTGCGGTA
PXO99A gene	primer-F	primer-R
PXO_RS00410	ATCAGCCGATCTTTATAACCTCT	TTTGGCCTTCATTGACGAAA
PXO_RS00385	AACACGCGGATCGACAAT	ATAATTCCGACCATCCTGACC
PXO_RS00405	GAATCGTTCGTTATCCAGCAATA	GATTGGCTGACTCAAGTTCGAC
PXO_RS00325	TCACTCAGGATATGAGCAAGATTC	GTCATGCAGGACGTTGAAGT
PXO_RS17595	AGCTGATCACCATCAACATGAG	AACACTTGGAAGAACATCTCCAG
PXO_RS00580	CCTTTTCCGATTCGCATTT	CAGGTCGTACATATAGGTGGTGAA
16S rRNA	ATGAAGTCGGAATCGCTAGTAATC	GATACGGCTACCTTGTTACGACTT
