# Supplementary figures and images for: OsNPR1 Enhances Rice Resistance to Xanthomonas oryzae pv. oryzae by Upregulating Rice Defense Genes and Repressing Bacteria Virulence Genes
Source: Int J Mol Sci. 2023 May 12;24(10):8687. doi: 10.3390/ijms24108687 (PMC10218434; doi:10.3390/ijms24108687)

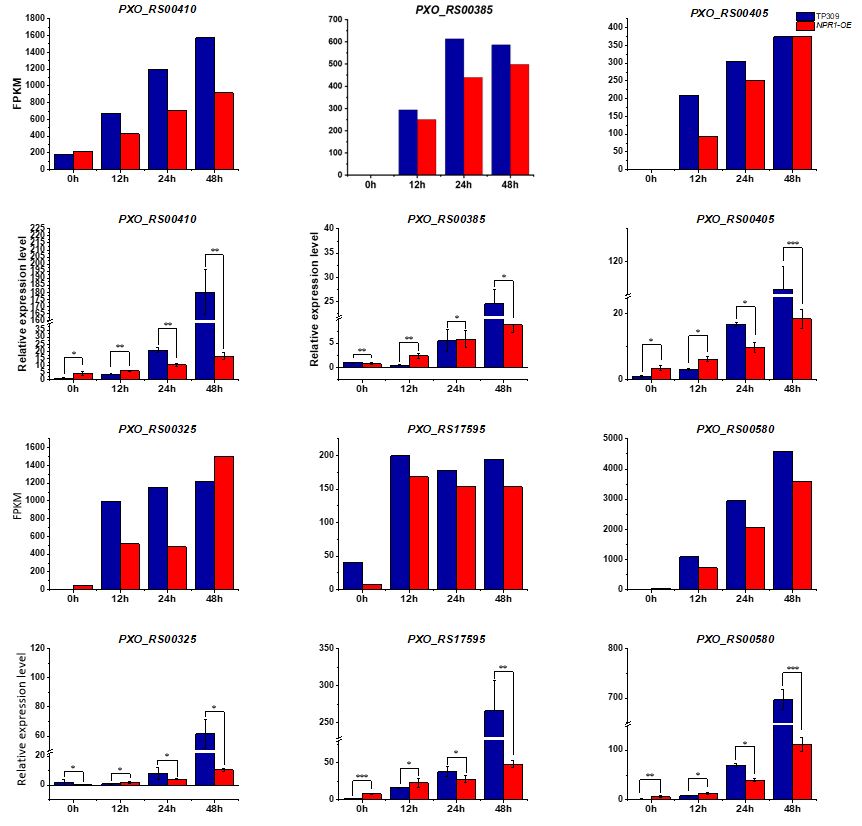

Supplement: Supplementary file 1 [file ijms-24-08687-s001.zip › Figure S1.JPG]
